# Establishing an international awareness day for paediatric rheumatic diseases: reflections from the inaugural World Young Rheumatic Diseases (WORD) Day 2019

**DOI:** 10.1186/s12969-020-00465-2

**Published:** 2020-09-11

**Authors:** Eve M. D. Smith, Sammy Ainsworth, Michael W. Beresford, Veerle Buys, Wendy Costello, Yona Egert, Helen E. Foster, Lovro Lamot, Berent J. Prakken, Christiaan Scott, Simon R. Stones

**Affiliations:** 1 Alder Hey Children’s NHS Foundation Trust, Liverpool Health Partners UK, Liverpool, UK; 2 Paediatric Rheumatology European Society, Geneva, Switzerland; 3 Department of Women and Children’s Health, Institute of Translational Medicine, Liverpool, UK; 4 European Network for Children with Arthritis, Geneva, Switzerland; 5 NIHR Alder Hey Clinical Research Facility, Liverpool Health Partners UK, Liverpool, UK; 6 Ouders van ReumaKinderen en –Adolescenten, De Haan, Belgium; 7 Certified patient expert, ReumaNet, Zaventem, Belgium; 8 Irish Children’s Advisory Network (iCAN), Tipperary, Ireland; 9 Inbar, Ramat Gan, Israel; 10 Great North Children’s Hospital, Newcastle Hospitals NHS Foundation Trust, Newcastle upon Tyne, UK; 11 Paediatric Global Musculoskeletal Task Force, Newcastle, UK; 12 Newcastle University Medicine Malaysia, Iskandar Puteri, Johor Malaysia; 13 Sestre Milosrdnice University Hospital Center, Zagreb, Croatia; 14 School of Medicine, University of Zagreb, Zagreb, Croatia; 15 Department of Pediatric Immunology, University Medical Center Utrecht, Utrecht, Netherlands; 16 Paediatric Rheumatology, University of Cape Town/Red Cross War Memorial Children’s Hospital, Cape Town, South Africa; 17 School of Healthcare, University of Leeds, Leeds, UK; 18 Collaboro Consulting, Bolton, UK

**Keywords:** Awareness, Campaign, Paediatric rheumatic diseases, WORD Day

## Abstract

There is a lack of awareness of paediatric rheumatic diseases (PRDs), among the public, and certain groups of healthcare professionals (HCPs), including general practitioners. To help improve international awareness and understanding of PRDs, World yOung Rheumatic Diseases (WORD) Day was established on 18 March 2019. Its aim was to raise awareness of PRDs and the importance of timely referral plus early diagnosis and access to appropriate treatment and support. A steering committee was established, and an external agency provided digital support. A social media campaign was launched in December 2018 to promote it, and analytics were used to measure its impact. Face-to-face and virtual events took place globally on or around WORD Day 2019, with 34 countries reporting events. Examples included lectures, social gatherings and media appearances. A total of 2585 and 660 individuals followed the official Facebook and Twitter accounts respectively, up until WORD Day. The official #WORDDay2019 hashtag was seen by 533,955 unique accounts on 18 March 2019 alone, with 3.3 million impressions. WORD Day 2019 was the first international campaign focused solely on PRDs. It demonstrated that despite awareness events being often resource-light, they can be implemented across a range of diverse settings. WORD Day has now become an annual global awareness event, facilitated by a growing network of patient, parent and professional community supporters.

## Background and purpose

Paediatric rheumatic diseases (PRDs) encompass a spectrum of conditions and affect children and young people (CYP) in many ways at a crucial time in their lives. The twenty-first century has seen the emergence of highly effective treatments [1], an increasing evidence base and greater understanding of diseases [2]. However, delays in diagnosis and access to appropriate care remain serious issues [3] and are reported around the world [4–6]. The reasons for the delay are multifactorial and include lack of awareness, referral pathways, cultural factors, geographical factors and inadequate capacity of the workforce. There is a lack of awareness amongst members of the public, and certain groups of healthcare professionals, including in particular general practitioners [7] and other primary healthcare providers such as nurses and physiotherapists, who are often the first point-of-contact for families. World yOung Rheumatic Diseases (WORD) Day (www.wordday.org) was established to improve global awareness and developed by the Paediatric Rheumatology European Society (PReS) (www.pres.eu) and the European Network for Children with Arthritis and Autoinflammatory Diseases (ENCA) (www.enca.org). It is one of the key PReS 2025 initiatives, in partnership with ENCA.

### Conceptualising and developing WORD Day

#### Establishing a steering committee

A steering committee, supported by PReS, consisted of a paediatric rheumatologist (ES), parents and carers of CYP (SA, VB, WC, YE), and a young person with experience of PRDs (SS), and met monthly via teleconference. The aim was to raise awareness to a wide international audience including the public, healthcare professionals (HCPs) and teachers, emphasising the importance of timely diagnosis and early access to treatment to control symptoms and impact on CYP’s lives.

#### Strategic planning

The steering committee, with input from ENCA and PReS, developed a WORD Day publicity strategy. They then worked closely with an external agency, MCI, to promote and disseminate the strategy. This began with a cost estimate accounting for an audit of comparable awareness campaigns, social media marketing and the promotion of WORD Day to the global rheumatology community. Branding guidelines were developed to ensure WORD Day materials were consistent with a colour palette, typeface and logo.

#### Communications and marketing

**Fig. 1:**
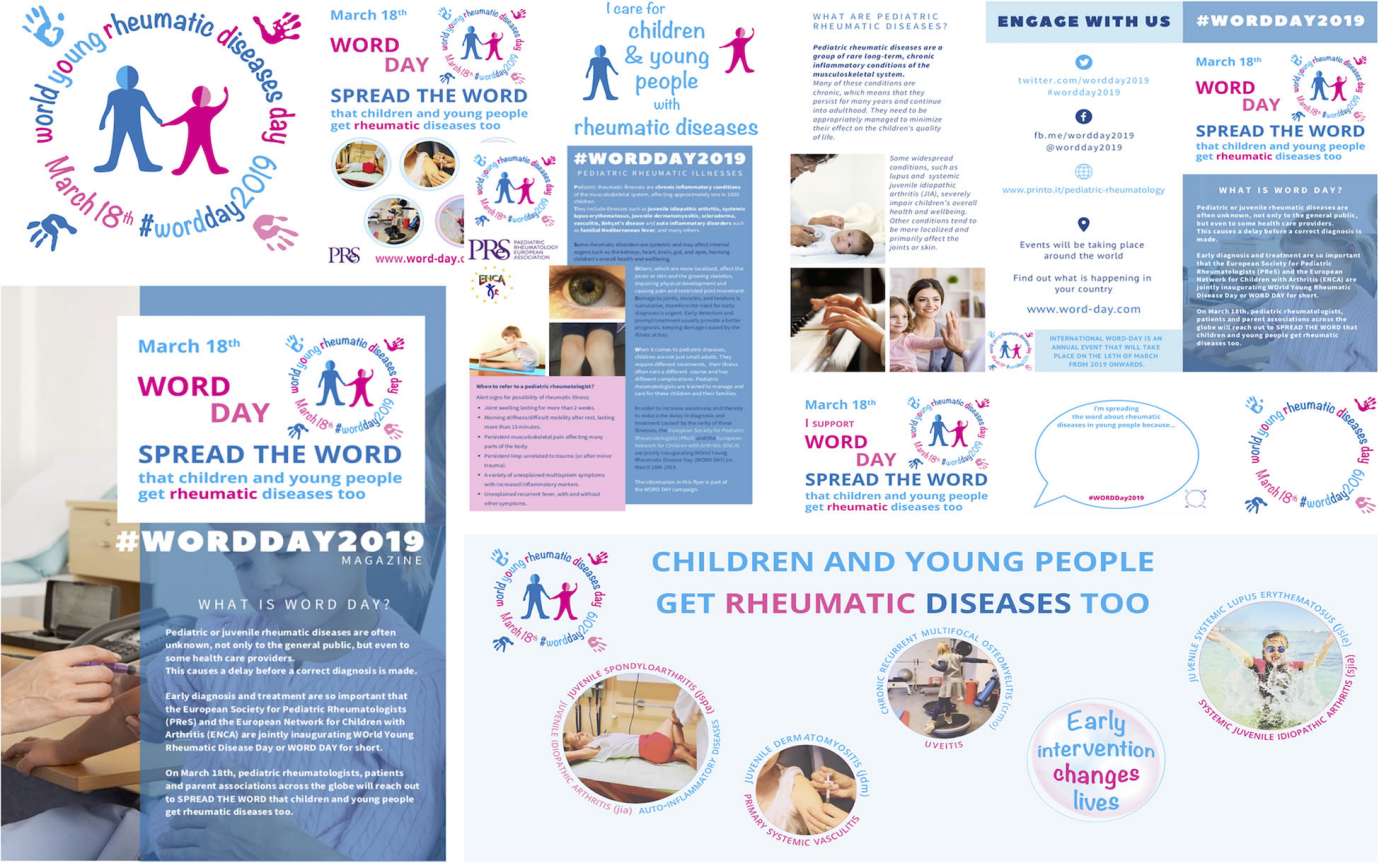
WORD Day branded marketing materials

Social media accounts (Facebook: www.facebook.com/wordday.org and Twitter: www.twitter.com/wordday_org) were created and information provided on the PReS website (see above). A range of branded materials were developed and approved, including logo, social media banners and filters, flyers, magazines, and a notice for healthcare professionals. These were all available free of charge for the global community to use and personalise (Fig. 1). An official campaign video was also created (http://vimeo.com/318626431). In addition, a public engagement activity was launched, titled the #ButtonChallenge2019. Participants were challenged to button up a piece of clothing while wearing gloves; the intention being to simulate the difficulties that CYP face when performing day-to-day activities with inflamed and painful hands. A range of additional awareness-raising activities were suggested, including awareness events (such as information stalls, coffee mornings, radio broadcasts and school assemblies) and educational/training programmes (for CYP, families and HCPs). Finally, individuals and organisations were encouraged to write blogs, create podcasts and share videos on living with PRDs.

#### Dissemination strategy

The primary international networks used to disseminate information about WORD Day were PReS and ENCA. The steering committee established links with various local and international organisations, both professional and patient/parent led, to aid global awareness. Communications included email, social media and posts published on the Paediatric Global musculoskeletal Task Force and other relevant social media pages. A special WORD Day edition of the PReS newsletter was also disseminated to the international PReS community.

### Activities which took place on WORD Day

**Fig. 2:**
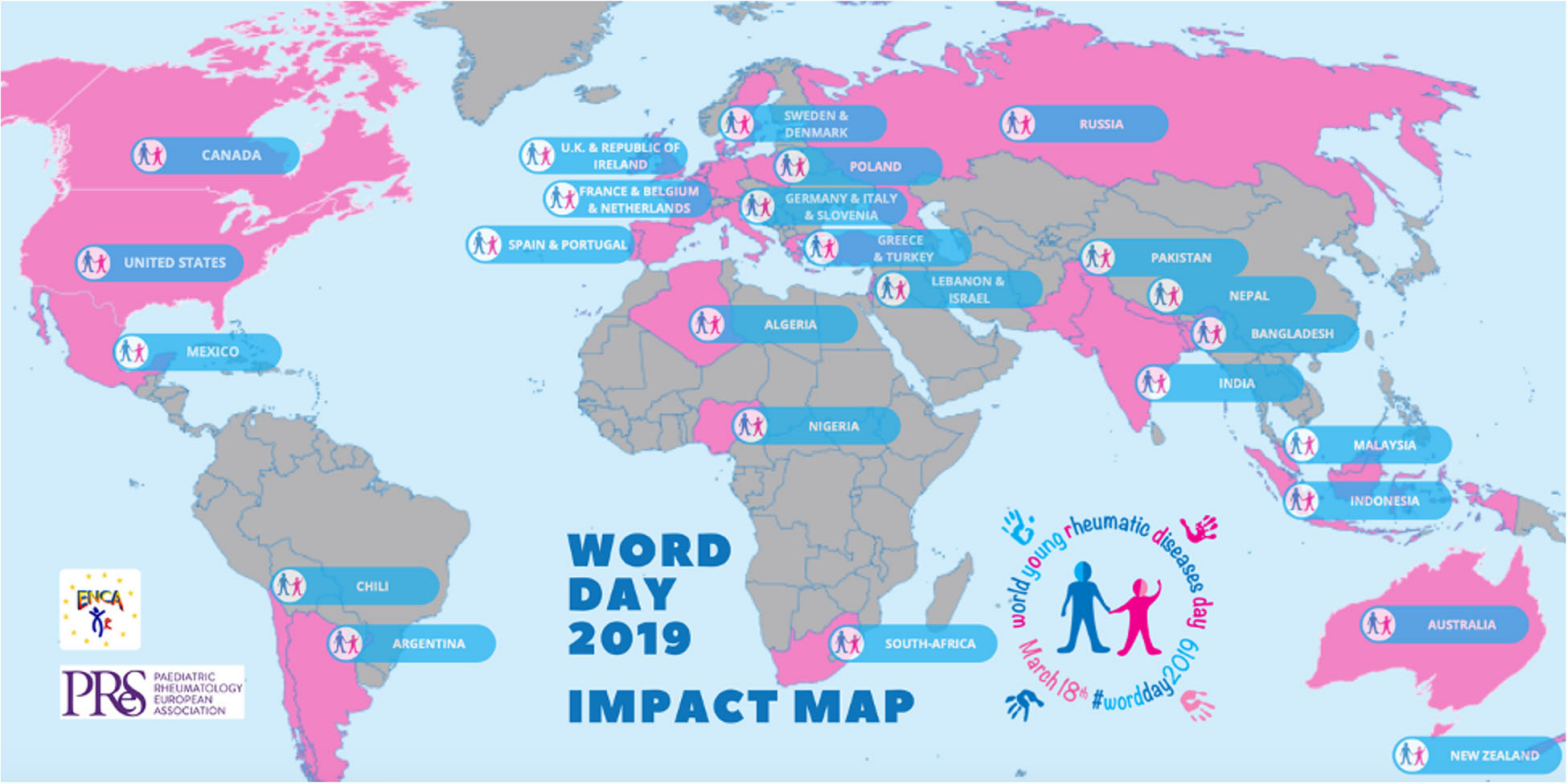
Impact map of WORD Day 2019 events around the world

**Fig. 3:**
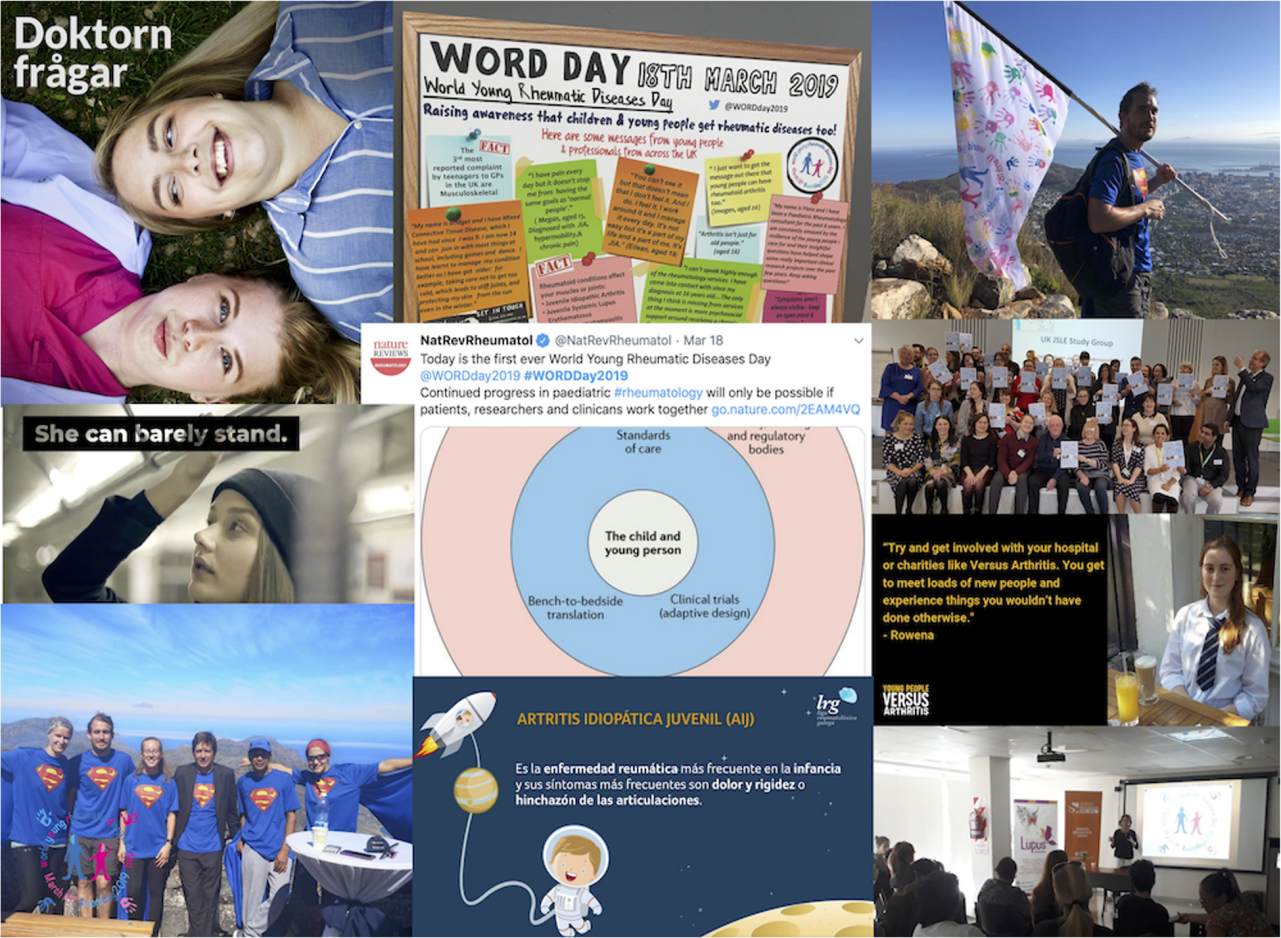
Selection of images published on social media on WORD Day 2019

A combination of face-to-face and virtual events took place around the world on or around WORD Day on 18 March 2019 (Table 1, Figs. 2 and 3), with 34 countries reporting events [8].

**Table 1:** Summary of WORD Day activities

WORD Day activities	Purpose
**Lectures to HCPs**	To improve awareness amongst general practitioners, paediatricians and other sub-specialists who may be the first people to come into contact with CYP presenting with PRDs.
**Social media live sessions, posts and tweet chat**	To discuss key challenges, examples of good practice and strategies for raising awareness with CYP, families, HCPs, patient/parent and professional organisations.
**Uplighting of monuments in WORD Day colours**	To improve awareness amongst the general public.
**Sponsored physical activities**	To improve awareness amongst the general public and achieve media coverage.
**Awareness stands and selfie booths**	To improve awareness amongst the general public through a presence in public places e.g. hospitals, universities and town centres, and by encouraging posts on social media displaying the WORD Day messaging.
**Appearances on television, radio and podcast**	To improve awareness amongst the general public, often featuring individual stories from CYP.
**Cake sales and coffee mornings**	To improve awareness of the general public and raise funds for local organisations.
**CYP and family gatherings and workshops**	To provide peer support and education, and information on research.

*CYP* Children and young people, *HCPs* Healthcare professionals, *WORD* World Young Rheumatic Diseases

### The social media campaign

**Fig. 4:**
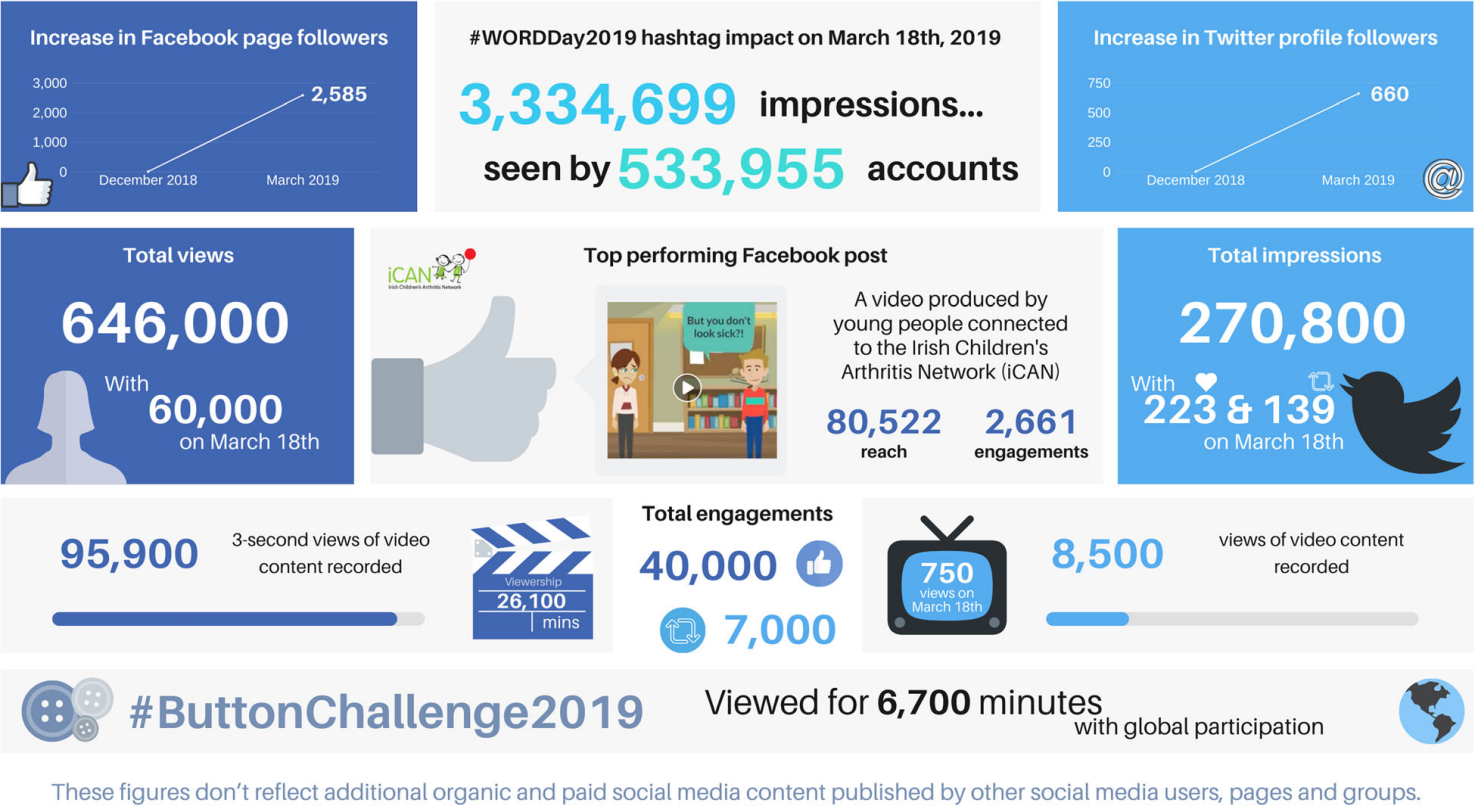
Social media analytics of the #WORDDay2019 hashtag

Between the launch of the WORD Day campaign on 21 December 2018 and WORD Day itself on 18 March 2019, both Facebook and Twitter platforms grew appreciably in terms of reach and engagement. Analytics were used to measure the impact of official WORD Day social media platforms (Fig. 4). Compared to digital marketing performance benchmarks in healthcare [9], and accounting for the size of audience, the average engagements per post for WORD Day-related content was greater than other medical-related accounts [9]. Targeted, funded promotions were also used on both platforms. In order to maintain an engaged audience, social media platforms needed to be kept active on an ongoing basis, particularly on Facebook, since this emerged as the most popular platform. Video content featuring those living with PRDs prevailed as a popular publication, particularly with authentic posts featuring CYP with PRDs. In addition, to add value for audiences, commissioned posts could be included in the future to incentivise certain groups of users to engage with content. For example, general lifestyle blogs for CYP and families, and accessible factsheets for HCPs to use in clinical practice. In addition, the #ButtonChallenge could be repeated year-on-year to leverage previous participation and grow the reach of the challenge. Overall, the 2019 campaign demonstrated that organic social media content is ideal for building communities and a shared vision, while funded social media content can reinforce core messaging shared via organic posts. With WORD Day, listening and learning from users around the world who were promoting, and engaging in WORD Day activities was essential for building visibility and a global sense of ownership.

## Conclusions

WORD Day 2019 was the first international campaign specifically focused on CYP with PRDs. It began as a simple idea amongst a small group of HCPs, CYP and parents, and grew internationally as a unique awareness day to amplify the voice of CYP with PRDs and their families. Through clear and open communication, individuals and organisations around the world were inspired to take action. Organic and funded social media content further aided the dissemination of the WORD Day message, with Facebook proving to be a popular platform to disseminate messages. Despite a wealth of different content published, authentic materials, namely videos proved to be the most popular with users, particularly when featuring material designed by and with CYP.

WORD Day has now become an annual global awareness event taking place on March 18th, facilitated by a growing network of patient, parent and professional community supporters.

## Data Availability

Not applicable.

## Abbreviations

CYP: Children and Young People
ENCA: European Network for Children with Arthritis and Autoinflammatory Diseases
PRD: Paediatric Rheumatic Diseases
PReS: Paediatric Rheumatology European Society
WORD: World yOung Rheumatic Diseases
